# Information-Seeking Patterns and Communication Preferences Among Japanese Survivors With Cancer: Cross-Sectional Analysis

**DOI:** 10.2196/79065

**Published:** 2026-05-28

**Authors:** Manae Hirayama, Daisuke Kasai, Ken-ichi Tabei

**Affiliations:** 1 Graduate School of Industrial Technology, Advanced Institute of Industrial Technology, Tokyo Metropolitan Public University Corporation, 1-10-40, Higashiooi, Shinagawa, Tokyo, 140-0011, Japan, 81 3 3472 7831

**Keywords:** patient preference, latent class analysis, survivor with cancer, mental health, patient-centered communication, theory of planned behavior

## Abstract

**Background:**

With increasing numbers of survivors with cancer, the importance of patient-centered information provision and communication to alleviate psychological burdens, such as anxiety and depression, is growing. However, substantial individual differences exist in patients with cancer information–seeking behaviors and use of support services, and few studies have comprehensively examined cognitive and psychological factors such as treatment status, sex, trust in information sources, and patient-provider relationships.

**Objective:**

This study aimed to integrate the theory of planned behavior and the patient-provider relationship model to identify latent subgroups among Japanese survivors with cancer using information-seeking behaviors, difficulties in information seeking, trust in information sources, and intentions to use psychosocial support services recommended by medical institutions.

**Methods:**

A CHERRIES (Checklist for Reporting Results of Internet E-Surveys)–compliant cross-sectional web survey was conducted in December 2024 with 350 Japanese survivors with cancer (at least 1 year post diagnosis, either undergoing treatment, or within 5 years after completing treatment). Exploratory factor analysis examined items such as difficulties in information seeking, trust in information sources, and assessment of relationships with physicians. Using the resulting factor structure and sociodemographic and clinical characteristics, latent class analysis was conducted. Differences between classes were examined using the chi-square test, Kruskal-Wallis test, and post hoc analyses.

**Results:**

Latent class analysis classified participants into 3 groups: women under observation, men under observation, and patients under treatment. In the women under observation group, evaluation of the reliability of information from nonmedical institutions was significantly higher than in the male group (*χ*²_₂_=12.30; *P*=.002). Although information-seeking behavior among men under observation was relatively limited, their evaluation of relationships with physicians was significantly higher than that of the treatment group (*χ*²_₂_=12.20; *P*=.002). The proportion of men who regarded promoting communication with doctors and health care professionals as a benefit of using support services was also significantly higher than that of women (*χ*²_₂_=12.57; *P*=.002 and Class 2 > Class 1; *P*=.001). In the treatment group, searches for information on life during treatment (*χ*²_₂_=7.22; *P*=.03), use of the Cancer Consultation Support Center (*χ*²_₂_=17.31; *P*<.001), and use of the National Cancer Center website (*χ*²_₂_=7.59; *P*=.02) were significantly higher than among men under observation. The treatment group also reported greater difficulty in seeking information (*χ*²_₂_=11.90; *P*=.003).

**Conclusions:**

Information-seeking behaviors, trusted sources, and perceived difficulties differed by sex and treatment stage among Japanese survivors with cancer. Patients undergoing treatment showed high information needs but greater difficulty in seeking information, suggesting reduced perceived behavioral control. Men under follow-up emphasized relationships with physicians, whereas women relied more on nonmedical information sources. These findings indicate that psychosocial support and information provision should be optimized according to patient-provider communication patterns.

## Introduction

Currently, approximately 20 million people worldwide are newly diagnosed with cancer each year [1], and this number is expected to reach 35 million by 2050 [2]. In Japan, the number of new patients with cancer is projected to reach approximately 4 million between 2035 and 2039 [3]. Owing to advances in treatment methods and early detection, many individuals diagnosed with cancer are now long-term survivors, and the number of people classified as survivors with cancer who live with and beyond cancer is increasing [4]. Regardless of treatment status, survivors with cancer tend to face various difficulties, including physical, mental, economic, social, and emotional challenges [5]. In particular, the prevalence of psychological stress, such as anxiety and depression, is high, making it extremely important to address their emotional needs [6,7]. Against this background, health care professionals are expected to provide appropriate information and high-quality communication so that patients can confidently make decisions about their diagnosis, prognosis, and treatment options [8].

The most frequently reported unmet nonmedical need among patients with cancer is information [9]. Accurate knowledge of the disease and its treatment affects psychological well-being, quality of life, and satisfaction with treatment [10]. However, the quality of information, access to it, and the individual’s level of understanding pose challenges. Improving the quality of the information provided and accurately identifying patient needs also leads to better communication between patients and physicians [11,12]. Nonetheless, many survivors with cancer report that the medical terms and explanations used by health care professionals are difficult to understand, resulting in insufficient information and communication [13,14]. Therefore, while patient-centered communication [15,16]—which enhances the reliability of information for survivors with cancer—is important, dissatisfaction may not stem solely from a simple lack of communication but may instead be intricately related to individual cognitive and psychological factors, which need to be elucidated.

Given this background, we conducted a pilot study using content analysis of free-text responses regarding the use of psychosocial support services among survivors with cancer aged 40 years and older [17]. The results suggested that factors such as trust in support providers, expectations for emotional regulation, difficulty in verbalizing emotions, a desire for independence, the personality of support providers, clarity of consultation procedures, and awareness and dissemination of services are related to the use of support services [17]. These findings suggest that the usage of psychosocial support services may be influenced not only by access and information deficiencies but also by individual cognitive and behavioral characteristics, including personal values. Thus, it is necessary to introduce a theoretical framework to comprehensively and quantitatively capture these complex factors.

The theory of planned behavior (TPB) has been widely used to explain behavior change among patients with chronic illnesses [18,19]. According to the TPB, behavioral intention is formed by 3 factors: “attitude,” “subjective norms,” and “perceived behavioral control” [20]. However, because the behavior of patients with chronic illnesses is influenced not only by internal cognitive factors but also by external factors such as the living environment, support networks, and health care systems, in this study we integrated external and relational factors, such as the living environment, support networks, and health care systems of survivors with cancer, into the TPB framework to deepen our understanding of the formation of behavioral intentions.

When addressing communication between patients and health care professionals, which is a central focus of this study, the patient-provider relationship model [21] is important. This model explains the impact of the relationship between health care professionals and patients on health outcomes, such as survival, treatment and recovery, relief of symptoms and distress, emotional well-being, and pain control. Communication functions are classified into relationship-oriented functions, which emphasize strengthening the therapeutic relationship and responding to emotions, and task-oriented functions, which involve exchanging information, decision-making, supporting self-management, and managing uncertainty [21].

Previous research has often examined information-seeking behaviors and the use of support services among survivors with cancer on the basis of single attributes, such as treatment phase or sex [22-24]. There have been limited attempts to comprehensively typify diverse individual behavioral characteristics and psychological factors and theoretically clarify their backgrounds. Therefore, in this study, we aimed to fill this gap by using latent class analysis (LCA) to identify behavioral patterns and by examining the characteristics of these patterns from the perspectives of the TPB and the relationship-oriented and task-oriented functions of the patient-provider relationship model.

On the basis of the above theoretical framework, we focused on the following two research objectives in this study:

1. To identify latent groups that can be typified from the viewpoint of the TPB regarding information-seeking behavior of survivors with cancer, trust in information sources, difficulties faced while seeking information, and use of psychosocial support services.
2. To examine how the identified latent groups are characterized from the perspectives of the relationship-oriented and task-oriented communication functions of the patient-provider relationship model and to clarify the contextual factors behind information-seeking behaviors and the use of support services.

## Methods

### Research Design

This cross-sectional survey was conducted on the basis of the CHERRIES (Checklist for Reporting Results of Internet E-Surveys) [25] (Checklist 1).

The questionnaire, created in Japanese, consisted of 21 screens and 52 items on the website and was designed to be answerable within 25 minutes (Multimedia Appendix 1). To prevent missing data, all questions were set as mandatory in the web-based questionnaire. To eliminate duplicate responses, any responses that matched IP address, sex, age, and place of residence were considered to be from the same respondent, and only the first response was included in the analysis.

Questions regarding patients with cancer information-seeking behavior were developed with reference to previous studies [26] that aimed to clarify the information needs and difficulties encountered when searching for information and using psychosocial support services among patients with ovarian cancer, as well as the patient experience survey [27] conducted by the National Cancer Center. During development, 3 researchers with experience in medical research and survey studies involving individuals with diseases examined whether each question appropriately reflected the research objectives and theoretical framework from the perspective of content validity and revised the questions for clarity of wording and to minimize respondent burden.

First, 12 items on sociodemographic information (including sex, age, place of residence, and occupation) and clinical information (including type of cancer, cancer stage, treatment status, and recurrence or metastasis) were collected using a single-choice format during the screening phase. This allowed for the collection of basic attribute information to uniquely classify each respondent. In addition, as a basic indicator related to information seeking, “device used to gather information” (1 item) was assessed using a single-choice format.

For information-seeking behavior regarding cancer, we started with the question “Have you ever searched for information about cancer?” Four multiple-choice checkbox items were used: “sources of cancer-related information,” “social networking service (SNS) or apps used for collecting cancer-related information,” and “advantages of psychosocial support and information-gathering services recommended by medical institutions.” For these questions, mutually exclusive options such as “did not search,” “do not wish to obtain information,” “do not use SNS or apps,” and “not applicable” were provided to ensure consistency in responses.

Furthermore, skip logic was introduced: if a respondent answered “did not search” to the question “Have you ever searched for information about cancer?” they were not required to answer questions about “sources of cancer-related information,” “devices used to collect information,” or “SNS or apps used for collecting cancer-related information,” and were instead directed straight to the question about “difficulties in information seeking.” Additionally, even if respondents had searched for information, if they selected either “do not wish to obtain information” or “do not use SNS or apps,” they were not asked to answer the question about the “device used to gather information.”

For the intention to use psychosocial support and information provision services recommended by medical institutions (corresponding to “behavioral intention” in the TPB), a yes or no question—“use of hospital-recommended apps and services for counseling support and information gathering”—was used (1 item). Only participants who answered “Yes” were able to proceed to the subsequent question on “advantages of psychosocial support and information-gathering services recommended by medical institutions.”

“Difficulties in information seeking” consisted of 7 items, “trusted information sources” consisted of 8 items, and “evaluation of interpersonal relationships between patients and health care providers” consisted of 9 items. To reduce the respondent burden, these were presented in a matrix format and measured using a 5-point Likert scale.

To assess personality traits, the Japanese version of the 10-Item Personality Inventory (TIPI-J) [28], which uses 10 items and a 7-point Likert scale, was used. The TIPI-J measures the Big Five personality dimensions—extraversion, agreeableness, conscientiousness, neuroticism, and openness—with 2 items each and has shown high internal consistency: extraversion (α=.92), agreeableness (α=.85), conscientiousness (α=.82), neuroticism (α=.91), and openness (α=.86) [28]. The TIPI-J has also been shown to be associated with the emotional and cognitive health of patients with brain tumors [29], making it a valid indicator for this study.

To ensure accessibility and usability, a test page simulating the survey environment was created before the actual survey to confirm that the question display and skip logic functioned accurately.

### Targeting and Survey Distribution

The survey was conducted privately by Asmarc Inc (a Japanese marketing research company) from December 10 to December 12, 2024. The company operates an online research panel with over 16 million registered members. After agreeing to the membership terms, registered members completed preliminary and final registration, during which they provided basic demographic information, such as age, sex, place of residence, employment status, and medical history.

The survey was conducted using the D-style web and Monitas platforms (Asmarc Inc) and was conducted using a list of available questionnaires. The eligibility criteria for this study were based on patient-reported outcome criteria for survivors with cancer 1‐5 years after diagnosis [30]. Participants were required to be between 20 and 89 years of age at the time of the survey, to have been diagnosed with cancer at least 1 year prior, and to be either currently undergoing treatment or within 5 years of completing treatment. The following exclusion criteria were applied: participants who reported their treatment status as “before cancer treatment” or “other,” those who had been diagnosed with cancer less than 1 year prior, and those for whom more than 10 years had passed since the end of treatment.

The sample size was calculated using cancer incidence statistics in Japan [31] as the reference population. Assuming a 90% CI and a 5% margin of error, the required sample size was estimated to be approximately 270 participants. This calculation was performed using the *pwr* package (version 1.3-0) in R. The *pwr* package was originally developed by Stéphane Champely and is currently maintained by Helios De Rosario. Considering a previous recommendation that at least 300 participants are desirable for conducting LCA [32], the minimum target sample size for this study was set at 300 participants.

### Statistical Analysis

In this study, only data for participants with responses to all questions were included, and statistical analyses were performed using R (version 4.3.2; R Foundation for Statistical Computing). Sociodemographic information was stratified on the basis of the use of psychosocial support services, specifically by responses of “Yes” or “No” to “use of hospital-recommended apps and services for counseling support and information gathering.” Descriptive statistics were calculated for each group. The sociodemographic information included sex, age, place of residence, occupation, and “device used to collect cancer information.” Cancer-related information consisted of “cancer stage,” “cancer type,” “presence or absence of recurrence,” “presence or absence of metastasis,” “current treatment status,” “time of cancer diagnosis,” and “time of cancer treatment completion.” Place of residence was recategorized into 6 categories: Hokkaido and Tohoku, Kanto, Chubu, Kinki, Chugoku and Shikoku, and Kyushu and Okinawa. To simplify the classification of cancer types, lung cancer and small cell lung cancer were grouped as lung cancer; esophageal, gastric, small intestine, and colorectal cancers were grouped as gastrointestinal cancer; liver, biliary tract, and pancreatic cancers were grouped as liver, biliary tract, and pancreatic cancer; kidney cancer and cancers of the renal pelvis and ureter and urethra, and bladder cancer were grouped as kidney, ureter, and bladder cancer; ovarian, endometrial, and cervical cancers were grouped as gynecologic cancer; leukemia, malignant lymphoma, and multiple myeloma were grouped as hematologic cancer; and brain tumors, head and neck cancer, thyroid cancer, cancer of unknown primary, skin cancer, and malignant melanoma were grouped as other. For information-seeking behaviors of patients with cancer, descriptive statistics were calculated regarding “having searched for information about cancer,” “sources of cancer information,” “SNS and apps used to gather cancer information,” and “benefits of using psychosocial support and information-gathering services recommended by medical institutions.” To understand the relational structure among items, analyses were conducted using tetrachoric correlations. Exclusive options such as “did not search” for “having searched for information about cancer,” “did not want to obtain information” for “sources of cancer information,” “do not use SNS or apps” for “SNS and apps used to gather cancer information,” and “not applicable” for “benefits of using psychosocial support and information-gathering services recommended by medical institutions” were excluded from the statistical analyses. For the TIPI-J, descriptive statistics were calculated, and the relational structure among the items was examined using polychoric correlations.

### Structural Validity of the Exploratory Factor Analysis

Exploratory factor analysis (EFA) was conducted according to the reporting standards proposed by Watkins [33]. EFA was chosen to identify the underlying factor structure. The measurement scales used were “behavioral barriers experienced when collecting information about cancer,” “reliable information sources,” and “relationships with doctors, medical professionals, and society.” Each item was rated on a 5-point Likert scale ranging from 1 to 5. The appropriateness of conducting EFA was confirmed using the Bartlett test of sphericity [34]. The criteria for determining the number of factors included the Kaiser criterion of eigenvalues greater than 1 [35] and the minimum average partial (MAP) method [36]. Eigenvalues exceeded 1 up to the fifth factor (fifth factor=1.26), and the MAP exhibited its lowest value (0.03) at the fifth factor; therefore, a 5-factor solution was adopted. To choose the factor extraction method, multivariate normality was examined using the Mardia test [37]. Because skewness (skewness=62.14; *P*<.01) and kurtosis (kurtosis=613.96; *P*<.01) were significant, multivariate normality was rejected, and a method that did not assume normal distribution was required. Moreover, because the items used in this study were ordinal scales based on a 5-point Likert scale, factor analysis using the minimum residual method with a polychoric correlation matrix, which is suitable for ordinal data, was adopted [38,39]. Oblimin rotation [40] was used for simplification and theoretical convergence. Because oblique rotation was used, pattern coefficients of ≥0.37 were judged to be theoretically meaningful loadings, and coefficients of ≥0.40 were considered the primary criterion [41] for substantial interpretation. Cross-loadings of ≥0.3 were examined for interpretative caution. Internal consistency was determined using Cronbach α and McDonald ω, and factors with a reliability of ≥0.7 [42,43] and theoretical significance were judged as appropriate. The analysis was conducted using the *psych* package (version 2.6.2) in R. The *psych* package was developed and is maintained by William Revelle [44].

### The LCA

In this study, we hypothesized that information-seeking behaviors and the use of psychosocial support services among survivors with cancer are not uniform and that multiple latent profiles exist. To identify these subgroups on the basis of statistical modeling, we used LCA [45]. Unlike traditional distance-based cluster analysis, LCA estimates latent structures using probabilistic models, allowing for objective model comparisons using information criteria, making it well suited for identifying latent profiles [46]. The analysis followed the reporting standards of Weller et al [47], and the optimal number of latent classes was determined on the basis of statistical fit indices and theoretical interpretability [48]. For the measurement variables, we recoded variables to ensure estimation stability, categorizing age, occupation, treatment status, and information-gathering devices. To examine the indicators included in the LCA, we conducted a polychoric correlation analysis and included 10 variables—sex, age, employment status, cancer type, stage, recurrence, metastasis, treatment status, time since completing treatment, and information-gathering devices—which were confirmed to have intervariable correlations of ±0.3 or higher. Model fit was assessed primarily using Bayesian information criterion (BIC), adjusted Bayesian information criterion (aBIC), and consistent Akaike information criterion (cAIC), and the model with the smallest value was selected as a candidate. Entropy was checked, with ≥0.8 considered acceptable and ≥0.9 considered ideal [48]. For the final model selection, we carefully evaluated whether each class had a sample size of n>50 or >5% of the total sample and whether each class profile was clinically meaningful. The R *poLCA* package (version 1.6.0.1), developed by Drew A Linzer and Jeffrey B Lewis, was used for the analysis [49].

### Multiple Comparison Tests Between Identified Latent Classes

We examined the differences in each survey item among the latent classes. The variables compared included “counseling support and information gathering through apps and services recommended by the hospital,” “survey items related to cancer,” “sources of information about cancer,” “SNS and apps used to gather cancer information,” and “advantages of using psychosocial support and information-gathering services recommended by medical institutions,” as well as the factors identified in the EFA and the TIPI-J. To assess statistical significance, we adopted nonparametric testing methods with a significance level of *P*<.05. For binary items, we used the chi-square test for independence, and for ordinal items, we used the Kruskal-Wallis test. For items where significant differences were observed, we conducted residual analysis for binary items and the Wilcoxon rank-sum test (*P=*.02) for ordinal items as post hoc analyses using the Bonferroni correction and evaluated the association between the latent classes and each survey item.

### Ethical Considerations

This study was approved by the Research Safety and Ethics Committee of Tokyo Metropolitan University of Industrial Technology (approval no 23020). The survey was conducted in compliance with the Declaration of Helsinki and relevant ethical guidelines, and only those who provided informed consent participated in the study. The collected data were securely stored in a password-protected database that was accessible solely to the research team. Participants were compensated with reward points according to the regulations of each platform. The allocation and exchange of points were managed exclusively by the survey company, and the research team was not informed about individual point allocations. Participants who responded via the D-style web platform received 2 points for the preliminary survey and 15 points for the main survey, totaling 17 points upon completing both surveys. Participants who responded via the Monitas platform received 2 points for the preliminary survey and 9 points for the main survey, totaling 11 points upon completing both surveys.

## Results

### Participant Characteristics

**Figure 1.**
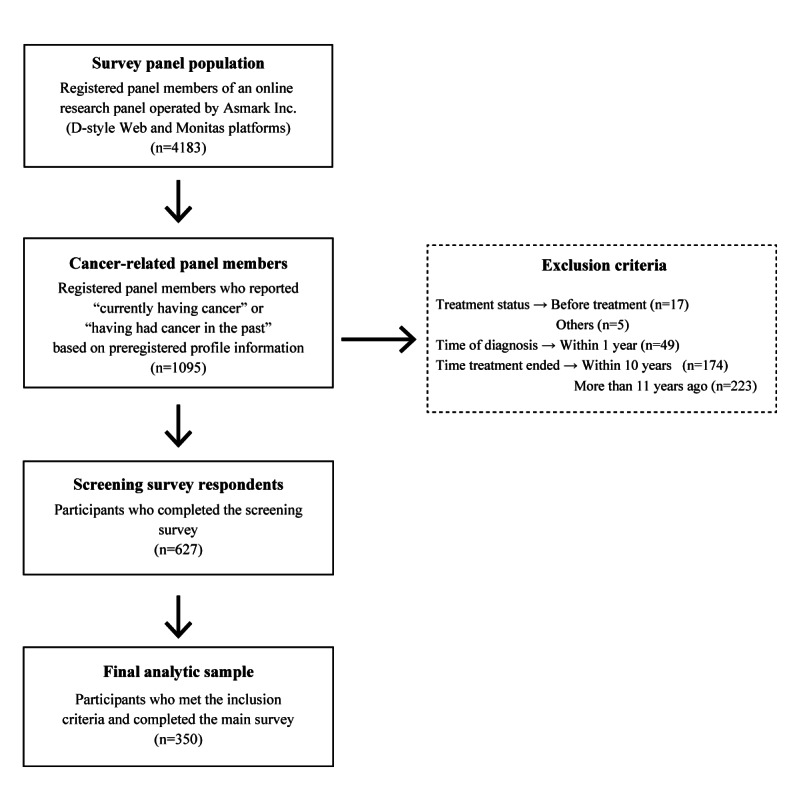
Flowchart of participant selection process.

Of the 4183 members registered on the D-style web and Monitas platforms, 1095 individuals who indicated in their preregistration information that they “currently have cancer or have had cancer in the past” were selected for the screening survey. Of these, 627 individuals responded (participation rate: 57.3%). Among the respondents, 350 met the eligibility criteria for the main survey and proceeded to participate (transition rate: 55.8%). Accordingly, the final analysis included 350 survivors with cancer (350/1095, 31.9%; Figure 1).

The survey participants comprised 175 men and 175 women (175/350, 50%). The average age was 57.9 (SD 10.8) years. The largest age group was 60‐69 years (138/350, 39.4%), followed by 70‐79 years (89/350, 25.4%). Regarding occupation, the most common response was unemployed (108/350, 30.9%), followed by company employees (84/350, 24%). Most participants resided in the Kanto region (156/350, 44.6%). Regarding clinical characteristics, the most common types of cancer were breast cancer (93/350, 26.6%) and gastrointestinal cancer (91/350, 26%). Many patients were at an early stage, with stage 1 accounting for 156 of 350 (44.6%) participants. Regarding treatment status, more than half had completed treatment and were undergoing regular follow-up visits at the hospital (202/350, 57.7%). Smartphones (193/350, 55.1%) were the most commonly used devices to gather information about cancer. In contrast, 105 of 350 (30%) participants reported that they did not search for information. Regarding the use of hospital-recommended apps and services for counseling support and information gathering, 282 of 350 (80.6%) participants answered “yes,” and 68 of 350 (19.4%) participants answered “no.” Among participants who reported that they did not search for information in response to the question about devices used to gather information about cancer (105/350, 30%), most also reported “yes” regarding the use of hospital-recommended apps and services for counseling support and information gathering (91/105, 86.7%), with only 14 of 105 (13.3%) reporting “no.” Of those who answered “yes,” 161 of 282 (57.1%) had completed treatment and were under regular follow-up, and 72 of 282 (25.5%) were receiving outpatient or home medical care. Smartphones were the most commonly used devices to gather information about cancer (161/282, 46%; Table 1).

**Table 1.** Patient characteristics (N=350).

Category	Total, n (%)	Yes, n (%)	No, n (%)
Use of hospital-recommended apps and services for counseling support and information gathering			
Sex			
Male	175 (50)	141 (40.3)	34 (9.7)
Female	175 (50)	141 (40.3)	34 (9.7)
Age (years)			
Mean (SD)	57.9(10.8)		
20‐29	3 (0.9)	3 (0.9)	0 (0)
30‐39	7 (2)	6 (1.7)	1 (0.3)
40‐49	32 (9.14)	28 (8)	4 (1.1)
50‐59	76 (21.7)	56 (16)	20 (5.7)
60‐69	138 (39.4)	114 (32.6)	24 (6.9)
70‐79	89 (25.4)	71 (20.3)	18 (5.1)
80‐89	5 (1.44)	4 (1.14)	1 (0.3)
Job			
Company employee	84 (24)	73 (20.9)	11 (3.1)
Civil servant	9 (2.6)	8 (2.3)	1 (0.3)
Self-employed	21 (6)	18 (5.1)	3 (0.9)
Company director	6 (1.7)	3 (0.9)	3 (0.9)
Freelance	6 (1.7)	4 (1.1)	2 (0.6)
Housewife and househusband	67 (19.1)	48 (13.7)	19 (5.4)
Part-time job	44 (12.6)	38 (10.9)	6 (1.7)
Unemployed	108 (30.9)	85 (24.3)	23 (6.6)
Other	5 (1.4)	5 (1.4)	0 (0)
Area			
Hokkaido and Tohoku	30 (8.6)	23 (6.6)	7 (2)
Kanto	156 (44.6)	129 (36.9)	27 (7.7)
Chubu	52 (14.9)	38 (10.9)	14 (4)
Kinki	68 (19.4)	53 (15.1)	15 (4.3)
Chugoku and Shikoku	22 (6.3)	19 (5.5)	2 (0.9)
Kyushu and Okinawa	22 (6.3)	20 (5.7)	2 (0.6)
Cancer type			
Lung cancer	29 (8.3)	24 (6.9)	5 (1.4)
Breast cancer	93 (26.6)	74 (21.1)	19 (5.5)
Gastrointestinal cancer	91 (26)	74 (21.1)	17 (4.9)
Liver, biliary tract, and pancreatic cancer	14 (4)	9 (2.6)	5 (1.4)
Kidney, ureter, and bladder cancer	17 (4.9)	14 (4)	3 (0.9)
Prostate cancer	39 (11.2)	31 (8.9)	8 (2.3)
Gynecological cancer	21 (6)	16 (4.6)	5 (1.4)
Blood cancer	30 (8.6)	27 (7.7)	3 (0.9)
Other	16 (4.6)	13 (3.7)	3 (0.9)
Stage			
Stage 1	156 (44.6)	129 (36.9)	27 (7.7)
Stage 2	87 (24.9)	67 (19.1)	20 (5.7)
Stage 3	68 (19.4)	54 (15.4)	14 (4)
Stage 4	39 (11.1)	32 (9.1)	7 (2)
Cancer recurrence			
Yes	62 (17.7)	55 (15.7)	7 (2)
No	288 (82.3)	227 (64.9)	61 (17.4)
Cancer metastasis			
Yes	55 (15.7)	47 (13.4)	8 (2.3)
No	295 (84.3)	235 (67.1)	60 (17.1)
Current treatment status			
Inpatient treatment	5 (1.4)	5 (1.4)	0 (0)
Outpatient or home visit treatment	87 (24.9)	72 (20.6)	15 (4.3)
Completed treatment and under regular observation	202 (57.7)	161 (46)	41 (11.7)
Completed treatment, no hospital visits	56 (16)	44 (12.6)	12 (3.4)
When was the cancer diagnosed?			
Within 3 years	127 (36.3)	109 (31.1)	18 (5.1)
Within 5 years	85 (24.3)	61 (17.4)	24 (6.9)
Within 10 years	96 (27.4)	75 (21.4)	21 (6)
>11 years	42 (12)	37 (10.6)	5 (1.4)
Time when cancer treatment ended			
During treatment	58 (16.6)	49 (14.0)	9 (2.6)
Within 1 year	114 (32.6)	96 (27.4)	18 (5.1)
Within 3 years	86 (24.6)	60 (17.1)	26 (7.4)
Within 5 years	92 (26.3)	77 (22.0)	15 (4.3)
Devices used to gather information about cancer			
Smartphone	193 (55.1)	161 (46.0)	32 (9.1)
Personal computer	12 (3.4)	10 (2.9)	2 (0.6)
Tablet (eg, iPad)	8 (2.3)	6 (1.7)	2 (0.6)
Other	32 (9.1)	14 (4)	8 (5.1)
Not searching for information	105 (30)	91 (26)	14 (4)

### Information-Seeking Behavior of Patients With Cancer

#### Topics Researched Regarding Cancer

The most frequently researched topic related to cancer was “my disease,” which accounted for 75.7% (265/350) of responses. This was followed by “about treatment” (68.6%, 240/350) and “progress and prognosis” (55.4%, 194/350). “Treatment costs” accounted for 38.9% (136/350) of responses, “life during treatment” for 36% (126/350), and “other patients’ treatment and daily life” for 27.1% (95/350). Regarding correlations between categories, “life during treatment” had a strong positive correlation with “about my own condition” (ρ=0.7) and “progress and prognosis” (ρ=0.64). “Other patients’ treatment and daily life” showed a moderate positive correlation with “about my own condition” (ρ=0.61).

#### Sources of Cancer Information and Use of SNS and Apps

The most common source of cancer-related information was “internet/SNS” (58.9%, 206/350), followed by “doctors, nurses, and other medical staff” (50.6%, 177/350). Other sources included “newspapers, magazines, and books” (17.7%, 62/350), “family, friends, and acquaintances” (15.7%, 55/350), “National Cancer Center website” (15.4%, 54/350), and “cancer counseling support center” (7.4%, 26/350). Regarding correlations between categories, a moderately strong correlation (ρ=0.69) was observed between “newspapers, magazines, and books” and “television and radio,” Among the SNS and apps used, YouTube (Google LLC) was the most common (13.4%, 47/350), followed by blogs (10%, 35/350), LINE, provided by LY Corporation (7.4%, 26/350), and X (formerly Twitter, provided by X Corp; 6%, 21/350). These items exhibited positive correlations with each other (ρ=0.48-0.6).

#### Benefits of Using Psychosocial Support and Information-Gathering Services Recommended by Health Care Institutions

The most commonly identified benefits of psychosocial support services or apps were “being able to obtain reliable information” (59.7%, 209/350) and “deepening knowledge about illness and treatment” (58.6%, 205/350), with more than half of the respondents emphasizing the quality and understanding of information. “Receiving information and support tailored to one’s own symptoms or situation” was cited by 45.4% (159/350) of patients, and “facilitating communication with doctors or medical staff” was cited by 29.4% (103/350) of patients. Regarding correlations between items, a strong positive correlation (ρ=0.71) was found between “deepening knowledge about illness and treatment” and “receiving information suited to one’s own symptoms or situation.” “Easy to operate and user-friendly” showed a moderate positive correlation with both “deepening knowledge about illness and treatment” (ρ=0.61) and “facilitating communication with doctors or medical staff” (ρ=0.6; Table 2 and Multimedia Appendix 2).

**Table 2.** Trends in information-seeking behavior among patients with cancer.

Item	Value, n (%)
Things I have researched about cancer	
About treatment	240 (68.6)
My disease	265 (75.7)
Course or prognosis	194 (55.4)
Information about other patients	95 (27.1)
Life during treatment	126 (36)
Cost of treatment	136 (38.9)
Other	20 (5.7)
Sources of cancer information	
Cancer Consultation Support Center	26 (7.4)
Doctors, nurses, and so on	177 (50.6)
Contact at public health center or health center	5 (1.4)
Newspapers, magazines, and books	62 (17.7)
Television and radio	40 (11.4)
National Cancer Center website	54 (15.4)
Internet and SNS^a^	206 (58.9)
Family, friends, and acquaintances	55 (15.7)
Other	6 (1.7)
SNS and apps used to gather cancer information	
Facebook (Meta Platforms, Inc)	14 (4)
Instagram (Meta Platforms, Inc)	15 (4.3)
LINE (LY Corporation)	26 (7.4)
X (formerly Twitter; X Corp)	21 (6)
YouTube (Google LLC)	47 (13.4)
Blog	35 (10)
Other	46 (13.1)
Benefits of using psychosocial support and information-gathering services recommended by health care institutions	
Access to reliable information	209 (59.7)
Easy to operate and use	76 (21.7)
Access to the latest medical information	152 (43.4)
Easy to understand correct usage and operation	78 (22.3)
Personal information is kept safe	58 (16.6)
Facilitate communication with doctors and medical staff	103 (29.4)
Gain knowledge about illnesses and treatments	205 (58.6)
Get information and support tailored to your symptoms and situation	159 (45.4)

a SNS: social networking services.

### TIPI-J

The mean scores reported by respondents were highest for neuroticism at 8.62 (SD 2.47), followed by openness at 8.24 (SD 2.43), extraversion at 7.65 (SD 2.6), and conscientiousness at 7.15 (SD 2.29). Agreeableness exhibited the lowest value among the 5 traits, at 5.84 (SD 2.03). A Shapiro-Wilk test [50] was conducted to assess the distribution of each scale, and the results were significant for extraversion (W=0.98; *P*<.001), agreeableness (W=0.95; *P*<.001), conscientiousness (W=0.98; *P*<.001), neuroticism (W=0.98; *P*<.001), and openness (W=0.98; *P*<.001), indicating that the data were not normally distributed. The correlations among the subscales were uniformly low (*r*=−0.43 to 0.37; Multimedia Appendix 2).

### EFA Results

The Bartlett test of sphericity was conducted to assess the applicability of factor analysis. On the basis of the results, the null hypothesis that the correlation matrix is an identity matrix was rejected (*χ*²_210_=5425.91; *P*<.001), indicating that there was a sufficiently robust correlation structure among the items. The Kaiser-Meyer-Olkin statistic was 0.81, confirming sampling adequacy and determining that factor analysis was appropriate. The number of factors was determined on the basis of eigenvalues, MAP, and theoretical consistency. Eigenvalues exceeded 1 up to the fifth factor (factor 1=6.33, factor 2=4.27, factor 3=2.56, factor 4=1.86, and factor 5=1.26), and the cumulative contribution of the 5-factor solution was 67.83%. MAP decreased with the addition of more factors and improved up to the fifth factor. In addition to these results, considering that the measurement included several conceptual domains such as “difficulties in information seeking,” “support and consultation for care,” “relationship with the physician,” “trust in information from medical institutions and specialized websites,” and “trust in information from outside medical institutions,” a 5-factor solution was adopted.

Factor 1 included items reflecting difficulties encountered in the process of seeking cancer-related information (factor loadings: 0.54-0.9) and was named “difficulties in information seeking.” Factor 2 included items related to continued employment before treatment, concerns about appearance, convalescence, precautions in daily life, and workplace considerations (factor loadings: 0.42-0.74) and was interpreted as “evaluation of support for cancer care.” Factor 3 included items on the primary physician’s knowledge and experience, ease of consultation, and trust in information provided by physicians (factor loadings: 0.67-0.81) and was named “evaluation of the relationship with the physician.” Factor 4 included trust in information from cancer-specialized or hospital or pharmaceutical company websites (factor loadings: 0.73-0.85) and was named “evaluation of the reliability of information from medical institutions and cancer-related websites.” Factor 5 included trust in sources of information outside medical institutions, such as family and acquaintances, other patients, and social networking sites (factor loadings: 0.47-0.76) and was named “assessment of the reliability of information from nonmedical institutions.”

The item “I had to visit several websites” loaded on both Factor 1 (0.60) and Factor 4 (0.33), and “the most reliable source is information from the physician” loaded on both Factor 3 (0.67) and Factor 4 (0.32). The internal consistency of the factors ranged from α=.75 to .9 and *ω*=0.78 to 0.92, with small interfactor correlations, the highest being between Factors 2 and 3 (*r*=0.47), justifying the treatment of the 5 factors as independent subconcepts (Table 3).

**Table 3.** Factor structure of information-seeking behavior and evaluation of information sources and psychosocial support services among survivors with cancer.

Item	Mean (SD)	Skewness	Kurtosis	Factor 1	Factor 2	Factor 3	Factor 4	Factor 5	H²
Factor 1: difficulties in information seeking									
I didn’t know where to look for it	2.88 (1.18)	0.08	−1.01	0.9	0.1	0	−0.15	0.01	0.82
I didn’t know who to ask	2.81 (1.18)	0.15	−1.03	0.88	0.04	−0.04	−0.09	0.05	0.8
I didn’t know what to look for	2.92 (1.17)	0.13	−0.96	0.88	0.05	0.03	−0.23	0.04	0.81
I couldn’t find the information I wanted right away	3.11 (1.14)	−0.16	−0.9	0.77	−0.01	0	0.12	−0.12	0.61
I didn’t know which information I could trust	3.04 (1.08)	−0.07	−0.8	0.71	−0.09	0.07	−0.01	0.05	0.51
I had to go to several websites	3.31 (1.09)	−0.43	−0.64	0.6	0	−0.04	0.33	−0.03	0.47
I couldn’t ask the doctor a question	2.25 (1.12)	0.73	−0.28	0.54	−0.1	−0.39	−0.01	0.06	0.57
Factor 2: support and consultation for care									
I think a medical professional spoke to me about continuing to work before starting treatment	3.15 (1.1)	−0.2	−0.54	−0.02	0.74	−0.06	0.1	0.02	0.55
I felt I could talk to a medical professional about my concerns about changes in appearance	3.07 (1)	−0.1	−0.31	0	0.73	−0.06	0.03	0.04	0.51
I think I was able to talk to someone about my illness and medical treatment	3.27 (0.97)	−0.24	−0.4	0.05	0.71	0.13	−0.03	−0.01	0.59
I think there were medical professionals other than my primary care physician whom I felt comfortable talking to.	3.58 (0.93)	−0.54	0.14	−0.04	0.62	0.29	0.06	−0.06	0.66
I think my workplace and work colleagues have made arrangements to allow me to continue both my treatment and work	3.02 (1.13)	−0.1	−0.55	−0.01	0.5	−0.09	0.08	0.08	0.26
I think I was able to get information from medical professionals about important points to keep in mind in daily life	3.38 (1.01)	−0.23	−0.66	0.03	0.48	0.17	−0.1	−0.09	0.31
I believe information about the patient’s treatment was shared among the medical professionals involved in the treatment	3.64 (0.86)	−0.34	−0.11	0.02	0.42	0.41	0.03	−0.02	0.51
Factor 3: relationship with the physician									
The primary physician has sufficient knowledge and experience about the patient’s cancer.	4.11 (0.88)	−0.9	0.43	−0.07	0.08	0.81	0.08	0.01	0.79
I think the primary physician who treated my cancer was easy to talk to.	3.97 (1)	−1.03	0.71	−0.05	0.16	0.8	−0.09	0.06	0.77
A reliable source of information is a doctor's information.	4.21 (0.77)	−0.94	1.26	0.03	0	0.67	0.32	0	0.64
Factor 4: trust in information from medical institutions and specialized websites									
Reliable sources of information include information from cancer-specific websites such as cancer information services.	3.68 (0.75)	−0.54	0.95	0.11	0.11	0.03	0.85	−0.01	0.79
Reliable sources of information include information from hospital websites.	3.63 (0.74)	−0.42	0.61	0.03	0.06	0.26	0.75	0.06	0.76
Reliable sources of information include information from pharmaceutical company websites.	3.29 (0.74)	−0.18	0.71	−0.04	0.13	0.01	0.73	0.09	0.63
Factor 5: trust in information from outside medical institutions									
Reliable sources of information include exchanging information with family and acquaintances.	2.99 (0.74)	−0.1	0.76	0	0	0.2	−0.12	0.76	0.57
Reliable sources of information include information shared among patients.	3.08 (0.76)	0.02	0.72	0.06	−0.09	−0.01	0.18	0.74	0.62
Reliable sources of information include information from lectures.	3.04 (0.71)	−0.34	1.44	0.05	0	0.01	0.36	0.61	0.58
Reliable sources of information included social media (Facebook and Instagram [Meta Platforms, Inc], X [formerly Twitter; X Corp], and YouTube [Google LLC]).	2.6 (0.78)	−0.45	−0.02	−0.04	0.1	−0.12	−0.09	0.47	0.25
Factor contribution	—^a^	—	—	4.32	4.26	4.03	3.09	2.03	—
Internal validity									
Crombach α	—	—	—	0.9	0.84	0.87	0.88	0.75	—
Macdonald ω	—	—	—	0.92	0.86	0.89	0.89	0.78	—
Factor correlation									
Factor 1	—	—	—	—	—	—	—	—	—
Factor 2	—	—	—	−0.08	—	—	—	—	—
Factor 3	—	—	—	−0.16	0.47	—	—	—	—
Factor 4	—	—	—	0.01	0.21	0.21	—	—	—
Factor 5	—	—	—	0.04	0.16	−0.07	0.18	—	—

a Not available.

### LCA Results

In determining the number of latent classes, models with 2-6 classes were estimated, and BIC was used as the primary index with comparisons to aBIC and cAIC. The 3-class model had the lowest BIC (5734.74) and cAIC (5805.74) and demonstrated high classification accuracy with an entropy of 0.95. Although the 4-class model had the lowest aBIC (5465) and similarly high entropy (0.96), both BIC and cAIC worsened (BIC=5766.37 and cAIC=5861.37). For models with 5-6 classes, the information criteria further increased, suggesting model overfitting. In finalizing the model, it was confirmed that each class had a sample size of n>50 or >5% of the total and that the class profiles were clinically interpretable. On the basis of minimum BIC and cAIC values and high classification accuracy, the 3-class model was selected as the final model (Table 4).

**Table 4.** Model selection criteria.

Model	Log-likelihood	Residual df	BIC^a^	aBIC^b^	cAIC^c^	Likelihood ratio	Entropy
Model 1	—^d^	—	—	—	—	—	—
Model 2	−2778.75	303	5832.82	5683.72	5879.82	1697.5	1
Model 3	−2659.41	279	5734.74	5509.5	5805.74	1458.82	0.95
Model 4	−2604.93	255	5766.37	5465	5861.37	1349.87	0.96
Model 5	−2582.65	231	5862.39	5484.88	5981.39	1305.3	0.94
Model 6	−2563.22	207	5964.12	5510.47	6107.12	1266.43	0.94

a BIC: Bayesian information criterion.

b aBIC: adjusted Bayesian information criterion.

c cAIC: consistent Akaike information criterion.

d Not available.

After fitting the model, the results for the 3 classes identified by the LCA were as follows. Class 1 comprised 95 individuals, all of whom were female (95/95, 100%). Most patients in Class 1 were between the ages of 50 and 69 years, with 35 of 95 (36.8%) aged 50‐59 years and 31 of 95 (32.6%) aged 60‐69 years. Regarding treatment status, 74 of 95 (77.9%) patients were undergoing regular follow-up after completing treatment, while 21 of 95 (22.1%) were in the follow-up stage without needing to visit a medical facility. On the basis of these treatment characteristics, this class was categorized as the “group of women under observation.” Breast cancer was the most common cancer type (52/95, 54.7%), followed by gynecological cancers (19/95, 20%). In terms of cancer stage, more than half of the patients were classified as stage 1 (56/95, 58.9%). Of the 95 patients, recurrence was observed in 8 (8.4%) and metastasis in 4 (4.2%). Regarding employment status, 51 of 95 (53.7%) patients were employed. For information-seeking behavior, 43 of 95 (45.3%) patients answered, “I do not use devices to gather information about cancer.” Among those who gathered information, smartphones were used by 39 of 95 (41.1%) patients; however, overall, information-seeking behavior was limited compared with that of patients in the other classes.

Class 2 comprised 163 individuals, the majority of whom were male (141/163, 86.5%). Most patients in Class 2 were aged 60 years or older, with 144 of 163 (88.3%) patients in this range; 76 of 163 (46.6%) patients were aged 60‐69 years, and 68 of 163 (41.7%) patients were ≥70 years. Regarding treatment status, 128 of 163 (78.5%) patients were under regular observation after completing treatment, while 35 of 163 (21.5%) were in the follow-up stage without requiring hospital visits. These features led to this class being categorized as the “group of men under observation.” The most common cancer was gastrointestinal cancer (69/163, 42.3%), followed by prostate cancer (29/163, 17.8%). Most cases were classified as early stage, with 76 of 163 (46.6%) patients at stage 1 and 37 of 163 (22.7%) patients at stage 2. Recurrence occurred in 21 of 163 (12.9%) patients, and metastasis occurred in 14 of 163 (8.6%) patients. These proportions were higher than those in Class 1 but lower than those in Class 3. Regarding employment, 92 of 163 (56.4%) patients were not employed. The device most commonly used for information seeking was a smartphone (104/163, 63.8%), and 31 of 163 (19%) patients answered “I do not use devices to gather information about cancer”; this proportion was lower than that among participants in the other classes.

**Figure 2.**
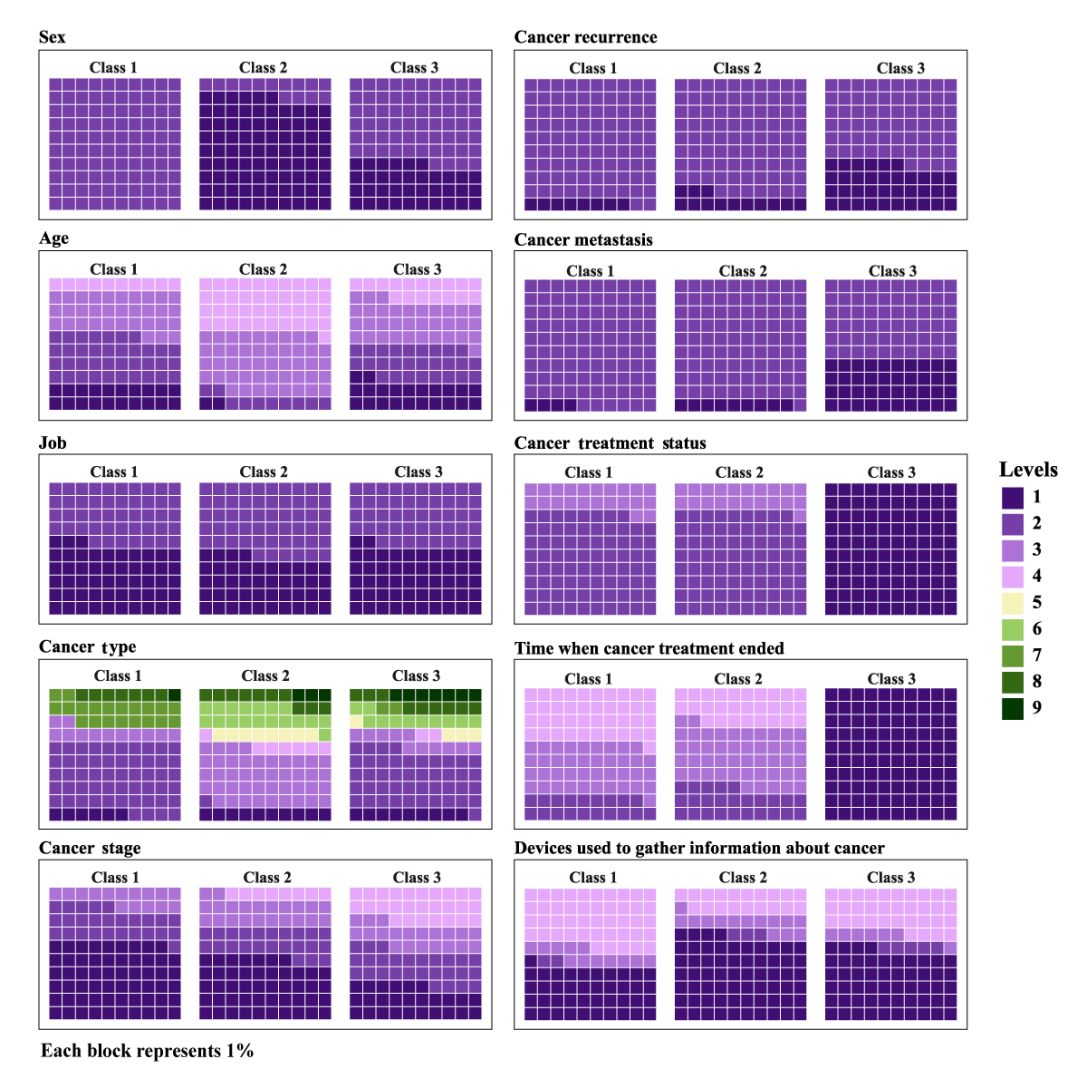
Class-specific conditional probabilities of sociodemographic and clinical characteristics identified by latent class analysis.

Class 3 comprised 92 individuals, all of whom were undergoing treatment (92/92, 100%). There were 59 (64.1%) females and 33 (35.9%) males, with the majority aged between 50 and 69 years (50‐59 years: 25/92, 27.2% and 60‐69 years: 31/92, 33.7%). Breast cancer was the most common cancer (41/92, 44.6%), followed by gastrointestinal (10/92, 10.9%) and prostate cancers (10/92, 10.9%). This class had a higher proportion of advanced cancers, with stage 4 accounting for 25 of 92 (27.2%) patients. Recurrence was observed in 33 of 92 (35.9%) patients and metastasis in 37 of 92 (40.2%) patients. Both proportions were higher in Class 3 than in the other 2 classes. Regarding information-seeking behavior, 50 of 92 (54.4%) patients used smartphones, but 31 of 92 (33.7%) patients answered “I do not use devices to gather information about cancer.” Given these features, this class was defined as the “group under treatment” (Figure 2 and Multimedia Appendix 2).

### Results of the Comparison Among the 3 Groups

**Figure 3.**
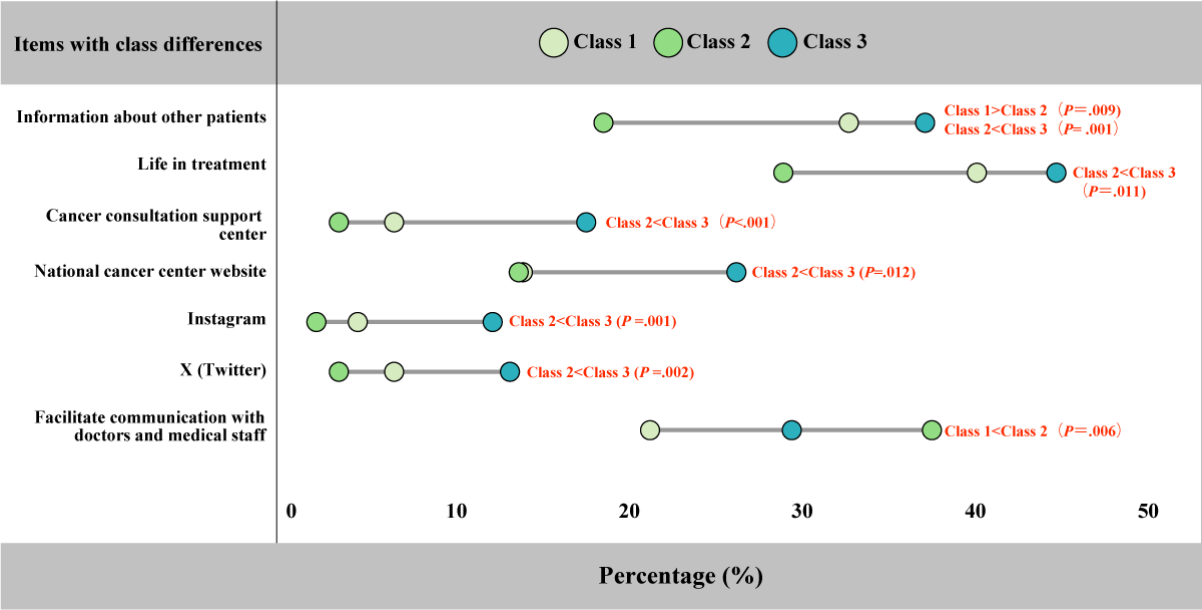
Results of multiple comparison analysis using the independent chi-square and post hoc tests.

All results were obtained from post hoc analyses. Regarding “use of hospital-recommended apps and services for counseling support and information gathering,” no significant differences were found. As shown in Figure 3, regarding “things I’ve researched about cancer,” the results revealed that “information about other patients” was significantly lower in Class 2 than in the other classes (*χ*²_₂_=12.22; *P=*.002, post hoc test: Class 1 > Class 2; *P*=.009, and Class 2 < Class 3; *P*=.001). Regarding “life during treatment” (*χ*²_₂_=7.22; *P*=.03 and post hoc test: Class 2 < Class 3; *P*=.01), Class 3 was significantly higher than Class 2. Regarding “sources of cancer information,” “Cancer Consultation Support Center” (*χ*²_₂_=17.31; *P*<.001 and post hoc test: Class 2 < Class 3; *P*<.001) and “National Cancer Center website” (*χ*²_₂_=7.59; *P*=.02 and post hoc test: Class 2 < Class 3; *P*=.01) were significantly higher in Class 3 compared with Class 2. Regarding “SNS and apps used to gather cancer information,” “Instagram” (*χ*²_₂_=12.57; *P*=.002 and post hoc test: Class 2< Class 3; *P*=.001) and “X (Twitter)” (*χ*²_₂_=9.55; *P*=.008 and post hoc test: Class 2 < Class 3; *P*=.002) were significantly higher in Class 3 compared with Class 2. Regarding “blog” (*χ*²_₂_=5.82; *P*=.054), no significant differences were found between classes after the post hoc test. For “benefits of using psychosocial support and information-gathering services recommended by health care institutions,” regarding “facilitate communication with doctors and medical staff” (*χ*²_₂_=12.57; *P*=.02 and post hoc test: Class 1 < Class 2; *P*=.006), Class 2 was significantly higher than Class 1 (Figure 3 and Multimedia Appendix 2).

**Figure 4.**
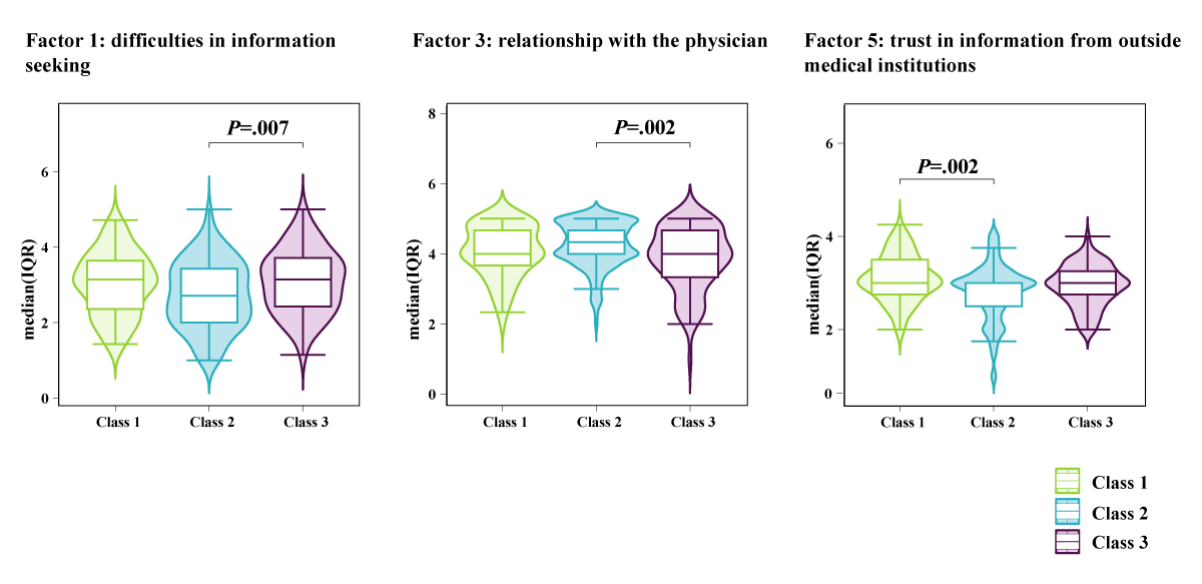
Results of multiple comparison analysis using the Kruskal-Wallis test and the Wilcoxon rank-sum test.

According to Figure 4, for “Factor 1: difficulties in information seeking” (*χ*²_₂_=11.9; *P*=.003 and post hoc test: Class 2 < Class 3; *P*=.007), Class 3 showed a significantly higher value than Class 2. For “Factor 3: evaluation of the relationship with the physician” (*χ*²_₂_=12.2; *P*=.002 and post hoc test: Class 2 > Class 3; *P*=.002), Class 2 had a significantly higher value than Class 3. For “Factor 5: assessment of reliability of information from nonmedical institutions” (*χ*²_₂_=12.3; *P*=.002 and post hoc test: Class 1 > Class 2; *P*=.002), Class 1 showed a significantly higher score than Class 2. Regarding the TIPI-J, agreeableness (*χ*²_₂_=6.7; *P*=.04) and neuroticism (*χ*²_₂_=8.8; *P*=.01) did not show significant differences after the post hoc test (Figure 4 and Multimedia Appendix 2).

## Discussion

### Principal Findings

The results revealed that age distribution and cancer type composition among the respondents in this study were generally consistent with trends in Japan’s national cancer statistics [31]. In addition, more than 80% of respondents indicated that they would like to use psychosocial support services or apps if recommended by medical institutions. Furthermore, in the results of the LCA, the “female under observation” group showed a high level of trust in “Factor 5: assessment of the reliability of information from nonmedical institutions,” whereas the “male under observation” group was less proactive in gathering information but placed a high value on “Factor 3: evaluation of the relationship with the physician” and “facilitate communication with doctors and medical staff,” indicating that they prioritize relationships with health care professionals. The “under treatment” group was the most active in collecting information, frequently using “information about other patients,” “life during treatment,” “Cancer Consultation Support Center,” and “National Cancer Center website.” However, the “Factor 1: difficulties in information seeking” score was also high, suggesting that treatment status and gender may influence barriers to gathering information and differences in trusted sources.

### Comparison With Previous Work

This study suggests that integrating the 3 factors from the TPB (ie, “attitude,” “subjective norms,” and “perceived behavioral control”) with the “relationship function” and “task-oriented function” from the patient-provider relationship model is associated with a stronger intention to use psychosocial support services and hospital-recommended apps. Within the TPB framework, hospital recommendations may consistently shape behavioral intentions as a form of subjective norm. Previous studies have also reported that recommendations from health care providers strongly influence information-seeking behavior and support service usage among patients with cancer [51], and the results of this study are consistent with these findings.

Looking at each group, the male group under follow-up showed a high evaluation of their relationship with physicians and a strong trust in physician-provided information, with subjective norms in the TPB being particularly dependent on physicians. Previous research has also shown that male patients with cancer place a high value on trustworthiness and sincerity in their interactions with health care providers [52]. Furthermore, in the male follow-up group, the relationship function based on trust in physicians appears to play a central role, possibly serving as a prerequisite for accepting psychosocial support services and information provision.

In contrast, the female group under follow-up showed relatively higher trust in “information from other patients” and nonmedical sources, suggesting that peer-derived experiential knowledge plays an important role in forming attitudes in the TPB. Earlier studies, particularly those focusing on patients with breast cancer, have reported that peer support contributes to acquiring information and alleviating anxiety about recurrence [53-55], which is consistent with the current findings. For this group, it is also possible that task-oriented functions, such as managing uncertainty and decision support, are supplemented outside health care institutions. By providing opportunities for exchange with other patients or sharing experiential information, health care providers may help translate behavioral intentions into action.

The “under treatment” group displayed the highest level of active information-seeking behavior among the 3 groups, but also the greatest difficulty in seeking information. In terms of the TPB framework, while “behavioral intention” to seek information or use psychosocial services was high, “perceived behavioral control” appeared to be low. From the perspective of the patient-provider relationship model, it was suggested that the task-oriented functions for exchanging information and managing uncertainty, and the relationship functions providing reassurance and emotional support, may not be sufficiently aligned with patients’ needs. Survivors with cancer undergoing treatment have strong information needs and clear intentions to seek information but may lack sufficient self-efficacy to understand the information and make decisions, leading to increased difficulty. Prior studies have also reported that patients with cancer in advanced or treatment phases experience heightened anxiety from online information [56], lack of social support beyond the internet, and negative treatment experiences [57]. Such negative experiences can decrease patients with cancer’s self-efficacy and trust in their digital skills, making information seeking a heavy burden. Therefore, for survivors with cancer undergoing treatment, clarifying reliable information sources and having health care providers organize and supplement information is crucial for alleviating the psychological burden associated with information-seeking.

### Strengths and Limitations

The strength of this study lies in its analysis of information-seeking behaviors of survivors with cancer and usage of psychosocial support services, grounded in the 3 components of TPB, as well as the communication functions of relationship-oriented and task-oriented functions in the patient-provider relationship model. By using EFA to identify factors and subsequently using LCA to classify multiple subgroups, this study systematically captured the behavioral intentions underlying information-seeking activities. This approach identified diverse needs among subgroups—needs that are often difficult to capture by a simple comparison of attributes—regarding trusted sources of information and communication tendencies among survivors with cancer. In particular, differences in the levels of trust in information sources between men and women during the follow-up period highlight the importance of personalized information provision, offering practical insights for future patient support service design. However, this study involved several limitations that should be considered. First, although the target sample size was achieved and trends were found to partially align with national cancer incidence statistics, the sampling was not stratified by age or cancer stage. Consequently, there was a bias toward survivors in stages 1 and 2, whereas the proportion of survivors in stages 3 and 4, or those who experienced recurrence or metastasis, was relatively low. Given the imbalances in cancer type, age group, and stage, caution is warranted regarding the generalization of the study findings to all survivors with cancer. Second, this was a cross-sectional study aimed primarily at identifying tendencies in types of information-seeking behaviors and thus cannot establish causal relationships related to various factors and backgrounds associated with each survey item. Detailed factor analyses and longitudinal studies are necessary to elucidate how information-seeking behaviors and perceptions evolve over time. Third, because this study focused on differences by treatment status and gender, socioeconomic factors such as income, education, and digital literacy were not included in the survey. However, these factors may also influence information-seeking behaviors and the use of support services, suggesting that future research should comprehensively examine these variables. Fourth, the questionnaire used in this study was developed on the basis of previous research and theoretical frameworks. However, because no formal pilot testing or test-retest reliability verification was conducted before the survey, the temporal stability of the measurement could not be assessed. This represents a limitation, and caution is required when interpreting the results. In the future, a comprehensive psychometric evaluation, including test-retest reliability and measurement invariance, will be necessary.

### Conclusions

This study clarified how information-seeking behaviors among survivors with cancer differ depending on treatment status and sex. By applying the TPB and patient-provider relationship models and using EFA, LCA, and multiple comparison analyses, we identified subgroups of patients with cancer with different behavioral characteristics. Our findings provide valuable insights for designing necessary approaches to promote personalized psychosocial support services and information provision for patients, as well as for improving the user interfaces of products and services that support patients psychologically and socially. Future research should focus on the realities of information provision and communication between patients with cancer and health care professionals, as they relate to individualized support for middle-aged and older survivors with cancer. Further exploration from multifaceted perspectives, including health care providers’ recognition of information delivery and patients’ acceptance of information, is expected to lead to the development and improvement of personalized and patient-centered communication approaches in the future.

## Supplementary material

10.2196/79065 Multimedia Appendix 1 Questionnaire survey form.

10.2196/79065 Multimedia Appendix 2 Datasets generated and analyzed during the study.

10.2196/79065 Checklist 1 CHERRIES checklist.

## Abbreviations

aBIC: adjusted Bayesian information criterion
BIC: Bayesian information criterion
cAIC: consistent Akaike information criterion
CHERRIES: Checklist for Reporting Results of Internet E-Surveys
EFA: exploratory factor analysis
LCA: latent class analysis
MAP: minimum average partial
SNS: social networking service
TIPI-J: Japanese version of the 10-item Personality Inventory
TPB: theory of planned behavior
