# Supplementary material for: Information-Seeking Patterns and Communication Preferences Among Japanese Survivors With Cancer: Cross-Sectional Analysis
Source: JMIR Cancer. 2026 May 28;12:e79065. doi: 10.2196/79065 (PMC13218279; doi:10.2196/79065)
Supplement: Multimedia Appendix 1 [file cancer-v12-e79065-s001.docx]

Questionnaire about yourself

SC1. What is your sex? (Choose one)

1 Male

2 Female

3 Other

SC2. What is your age? (Choose one)

1 19 years old or younger

2 20–29 years old

3 30–39 years old

4 40–49 years old

5 50–59 years old

6 60–69 years old

7 70–79 years old

8 80–89 years old

9 90 years old or older

SC3. Please tell us about your occupation. (Choose one)

1 Company employee

2 Government employee

3 Self-employed/private business

4 Company executive

5 Freelance

6 Housewife/househusband

7 Part-time/part-time job

8 Unemployed

9 Other

SC4. Please select the prefecture in which you live. (Choose one)

1 Hokkaido

2 Aomori Prefecture

3 Iwate Prefecture

4 Miyagi Prefecture

5 Akita Prefecture

6 Yamagata Prefecture

7 Fukushima Prefecture

8 Ibaraki Prefecture

9 Tochigi Prefecture

10 Gunma Prefecture

11 Saitama Prefecture

12 Chiba Prefecture

13 Tokyo

14 Kanagawa Prefecture

15 Niigata Prefecture

16 Toyama Prefecture

17 Ishikawa Prefecture

18 Fukui Prefecture

19 Yamanashi Prefecture

20 Nagano Prefecture

21 Gifu Prefecture

22 Shizuoka Prefecture

23 Aichi Prefecture

24 Mie Prefecture

25 Shiga Prefecture

26 Kyoto

27 Osaka

28 Hyogo Prefecture

29 Nara Prefecture

30 Wakayama Prefecture

31 Tottori Prefecture

32 Shimane Prefecture

33 Okayama Prefecture

34 Hiroshima Prefecture

35 Yamaguchi Prefecture

36 Tokushima Prefecture

37 Kagawa Prefecture

38 Ehime Prefecture

39 Kochi Prefecture

40 Fukuoka Prefecture

41 Saga Prefecture

42 Nagasaki Prefecture

43 Kumamoto Prefecture

44 Oita Prefecture

45 Miyazaki Prefecture

46 Kagoshima Prefecture

47 Okinawa Prefecture

SC5 Have you ever had cancer? (Choose one)

1 Yes

2 No

SC6. Please choose one type of cancer you have/had. (Choose one) (Please select the primary cancer. If you have/had multiple cancers, please answer the most recent type of cancer you have/had.)

1 Brain tumor

2 Head and neck cancer

3 Thyroid cancer

4 Lung cancer

5 Small cell lung cancer

6 Mesothelioma

7 Breast cancer

8 Esophageal cancer

9 Stomach cancer

10 GIST

11 Small bowel cancer

12 Colon cancer

13 Liver cancer

14 Biliary tract cancer

15 Pancreatic cancer

16 Kidney cancer

17 Renal pelvis, ureter, urethra

18 Bladder cancer

19 Prostate cancer

20 Ovarian cancer

21 Body cancer

22 Cervix Cancer

23 Skin cancer

24 Malignant melanoma (melanoma)

25 Neuroendocrine tumor (NET)

26 Sarcoma

27 Leukemia

28 Chronic lymphocytic leukemia (CLL)

29 Malignant lymphoma

30 Multiple myeloma

31 Myelodysplastic syndrome (MDS)

32 Myelofibrosis

33 Cancer of unknown origin

34 Other (rare cancer)

SC7. What is the stage of your cancer? (Select one) (If cancer has been cured, what was the stage of cancer at that time? If you have/had multiple cancers, please indicate the stage of the most recent cancer you had/have.)

1 Stage I

2 Stage II

3 Stage III

4 Stage IV

5 Don't know/not remember/other

SC8. Please tell us about your experience of recurrence/metastasis. (Select one for each)

|  | SC8-1. Have you ever had cancer recurrence? | SC8-2. Have you ever had cancer metastasis? |
| --- | --- | --- |
| 1 Yes | □ | □ |
| 2 No | □ | □ |

SC9. Please tell us about your current treatment status. (Choose one)

1 Before treatment

2 Undergoing inpatient treatment

3 Undergoing inpatient treatment (including home visits)

4 Treatment has been completed with regular hospital visits

5 Treatment has been completed and do not visit the hospital regularly

6 Other

SC10. Please tell us when you were first diagnosed with cancer. (Choose one)

1 Within the past 1 year

2 Within the past 3 years

3 Within the past 5 years

4 Within the past 10 years

5 More than 11 years ago

SC11. When did your cancer treatment end? (Choose one)

1 within 1 year

2 within 3 years

3 within 5 years

4 within 10 years

5 more than 11 years ago

SC12. Do you agree with the above information? (Choose one)

1 Agree

2 Disagree

Q1. When you researched about cancer, what kind of information did you research? (Multiple choice)

1 About Treatment

2 My Disease

3 Course/prognosis

4 Information about other patients

5 Life in treatment

6 Cost of treatment

7 Other

8 I didn't check

Q2. Where do you get information about cancer treatment and hospitals? (Multiple choice)

1 Cancer Consultation Support Center

2 Doctors, nurses, etc.

3 Contact at public health center/health center

4 Newspapers, magazines, books

5 Television/radio

6 National Cancer Center website

7 Internet/SNS

8 Family/friends/acquaintances

9 Other

10 I don't want to get information

11 No answer

Q3: Which device do you use most often to gather information about cancer? (Choose one)

1 Smartphone

2 Computer

3 Tablet (e.g., iPad)

4 Not researching anything

5 Other

Q4: What social networking services or applications do you use to gather information about cancer? (Multiple choice)

1 Facebook

2 Instagram

3 LINE

4 X (formerly Twitter)

5 YouTube

6 Blog

7 Other

8 Don't use social media or applications

Q5: Do you have any difficulties in gathering information about cancer? (Select one for each)

|  | Q5-1. I didn't know which information I could trust | Q5-2. I had to go to several web sites | Q5-3. I couldn't find the information I wanted right away | Q5-4. I didn't know what to look for | Q5-5. I didn't know where to look for it | Q5-6. I didn't know who to ask | Q5-7. I couldn't ask the doctor a question |
| --- | --- | --- | --- | --- | --- | --- | --- |
| 1 Not at all | □ | □ | □ | □ | □ | □ | □ |
| 2 Not very much | □ | □ | □ | □ | □ | □ | □ |
| 3 Neither | □ | □ | □ | □ | □ | □ | □ |
| 4 More or less applicable | □ | □ | □ | □ | □ | □ | □ |
| 5 Very applicable | □ | □ | □ | □ | □ | □ | □ |

Q6. What do you consider to be reliable sources of information? (Select one for each)

|  | Q6-1. Doctor (trusted source) | Q6-2. Hospital website (trusted source) | Q6-3. Oncology Web site (trusted source) | Q6-4. Pharmaceutical company website (trusted source) | Q6-5. Patient-to-patient information (trusted source) | Q6-6. Lecture Iinformation (trusted source) | Q6-7. Exchange of information with family and acquaintances (trusted source) | Q6-8.  SNS Social network services (Facebook, X [(Twitter)], Instagram,,  YouTube) (trusted source) |
| --- | --- | --- | --- | --- | --- | --- | --- | --- |
| 1 Very low | □ | □ | □ | □ | □ | □ | □ | □ |
| 2 Low | □ | □ | □ | □ | □ | □ | □ | □ |
| 3 Neither | □ | □ | □ | □ | □ | □ | □ | □ |
| 4 High | □ | □ | □ | □ | □ | □ | □ | □ |
| 5 Very high | □ | □ | □ | □ | □ | □ | □ | □ |

Q7. Would you be willing to use an app or online service that provides counseling support and information if it were recommended in your hospital? (Choose one)

1 Yes

2 No

Q8. What do you think are the advantages of using an application or online service for consultation support and information gathering? (Multiple choice)

1. Access to reliable information
2. Easy to operate and use
3. Access to the latest medical information
4. Easy to understand correct usage and operation
5. Your personal information is kept safe
6. Facilitate communication with doctors and medical staff
7. Gain knowledge about illnesses and treatments
8. Information and support that is appropriate for you
9. Get information and support tailored to your symptoms and situation
10. None apply

Q9. How did you feel about communicating with your surroundings? (Select one for each)

|  | Q9-1. I think the primary physician who treated my cancer was easy to talk to. | Q9-2. The primary physician has sufficient knowledge and experience about the patient’s cancer. | Q9-3. I think there were medical professionals other than my primary care physician whom I felt comfortable talking to. | Q9-4. I believe information about the patient's treatment was shared among the medical professionals involved in the treatment | Q9-5. I think I was able to get information from medical professionals about important points to keep in mind in daily life | Q9-6. I think a medical professional spoke to me about continuing to work before starting treatment | Q9-7. I felt I could talk to a medical professional about my concerns about changes in appearance | Q9-8. I think I was able to talk to someone about my illness and medical treatment | Q9-9. I think my workplace and work colleagues have made arrangements to allow me to continue both my treatment and work |
| --- | --- | --- | --- | --- | --- | --- | --- | --- | --- |
| 1 Not at all | □ | □ | □ | □ | □ | □ | □ | □ | □ |
| 2 Not very much | □ | □ | □ | □ | □ | □ | □ | □ | □ |
| 3 Neither | □ | □ | □ | □ | □ | □ | □ | □ | □ |
| 4 Somewhat | □ | □ | □ | □ | □ | □ | □ | □ | □ |
| 5 Very much | □ | □ | □ | □ | □ | □ | □ | □ | □ |

Q10. What do you think about your personality? (Select one for each)

|  | Q10-1. I think I am active and diplomatic | Q10-2. I think I am frustrated and quarrelsome with others | Q10-3. I think I am firm and strict with myself | Q10-4. I think I am anxious and easily upset | Q10-5. I think I like new things and have unusual ideas | Q10-6. I think I am reserved and quiet | Q10-7. I think I am a kind person who cares about others | Q10-8. I think I am sloppy and careless | Q10-9. I think I am calm and stable | Q10-10. I think I am ordinary and lacking in ideas |
| --- | --- | --- | --- | --- | --- | --- | --- | --- | --- | --- |
| 1 Disagree strongly | □ | □ | □ | □ | □ | □ | □ | □ | □ | □ |
| 2 Disagree moderately | □ | □ | □ | □ | □ | □ | □ | □ | □ | □ |
| 3 Disagree a little | □ | □ | □ | □ | □ | □ | □ | □ | □ | □ |
| 4 Neither agree nor disagree | □ | □ | □ | □ | □ | □ | □ | □ | □ | □ |
| 5 Agree a little | □ | □ | □ | □ | □ | □ | □ | □ | □ | □ |
| 6 Agree moderately | □ | □ | □ | □ | □ | □ | □ | □ | □ | □ |
| 7 Agree strongly | □ | □ | □ | □ | □ | □ | □ | □ | □ | □ |
