# Supplementary material for: Information-Seeking Patterns and Communication Preferences Among Japanese Survivors With Cancer: Cross-Sectional Analysis
Source: JMIR Cancer. 2026 May 28;12:e79065. doi: 10.2196/79065 (PMC13218279; doi:10.2196/79065)
Supplement: Checklist 1 [file cancer-v12-e79065-s003.docx]

| Checklist item | Explanation | Section |
| --- | --- | --- |
| Describe survey design | The required sample size was estimated based on cancer incidence statistics in Japan. Using the pwr package in R with a 90% confidence level and a 5% margin of error, the minimum sample size was calculated to be approximately 270 participants. Considering prior recommendations that at least 300 participants are required for latent class analysis, the target sample size was set at 300.  This study employed a cross-sectional web-based survey. Participants were cancer survivors aged 20–80 years who were at least 1 year post-diagnosis and within 5 years of completing treatment. Respondents were recruited from Asmarc Inc.’s research panels (“D-style web” and “Monitas”) using a closed, convenience sampling approach. | Targeting and Survey Distribution |
| IRB approval | This study was approved by the Research Safety and Ethics Committee of the Tokyo Metropolitan University of Industrial Technology (approval number: 23020). | Ethical Consideration |
| Informed consent | Before starting the survey, participants were informed about the purpose of the study, the survey duration (within 25 minutes), the types of data to be collected, where and how long the data would be stored, and the affiliation of the principal investigator on the screen. Participation began after the participants reviewed the information on the consent screen and pressed the consent button. | Ethical Considerations |
| Data protection | The collected data were anonymized and did not include any personally identifiable information. The survey responses were securely stored on the research company’s password-protected server, and researchers could only access them with limited authorization. | Ethical Considerations |
| Development and testing | Before distribution, the survey was tested in a staging environment to verify the display, skip logic, required response settings, and usability. | Research Design |
| Open survey versus closed survey | This was a closed survey. | Targeting and Survey Distribution |
| Contact mode | The initial approach to participants was conducted entirely via the Internet. Invitations to participate in the survey were presented to members registered with Asmarq Inc.’s research panel through online platforms (“D-style web” and “Monitas”). No contact was made by mail or telephone. | Targeting and Survey Distribution |
| Advertising the survey | The survey targeted members of Asmark Inc.’s research panel, and no public advertisements, banner postings, or announcements through offline media were made. | Targeting and Survey Distribution |
| Web/E-mail | This survey was conducted as a web-based survey. After logging into the survey platform, participants entered their responses online, and the data were automatically saved to the database. | Targeting and Survey Distribution |
| Context | This survey was conducted using the survey list pages for members of a dedicated research platform operated by Asmark Inc. (“D-style web” and “Monitas”). These websites are specialized platforms where registered panelists participate in market and academic research, and visitors access them primarily to participate in surveys. | Targeting and Survey Distribution |
| Mandatory/voluntary | This survey was voluntary and was completed by members registered on dedicated survey platforms (“D-style web” and “Monitas”). Members reviewed the information about the study displayed on the survey list page and participated by logging in and responding only if they wished to participate. | Targeting and Survey Distribution |
| Incentives | Participants in this survey were awarded panel points upon completion of their responses, in accordance with the survey panel rules of Asmark Inc. The awarding and exchange of points were centrally managed by the survey company, and the research team did not have access to the individual point allocations. Participants who joined via the D-style web platform received 2 points for the preliminary survey and 15 points for the main survey, totaling 17 points. Participants who joined via the Monitas platform received 2 points for the preliminary survey and 9 points for the main survey for a total of 11 points. | Ethical Consideration |
| Time/Date | The survey was conducted from December 10 to 12, 2024. | Targeting and Survey Distribution |
| Randomization of items or questionnaires | Neither the order of the questions nor the entire questionnaire was randomized. | Research Design |
| Adaptive questioning | Conditional branching was set to ensure that questions that were not applicable to respondents would be automatically skipped according to respondents’ attributes and the responses to screening questions. Therefore, only necessary questions were presented to each participant, which reduced the overall complexity of the survey and respondent burden. | Research Design |
| Number of Items | The number of items per page ranged from 1–15. All questions were mandatory, and participants could not proceed to the next page without answering all questions. Thus, all participants who started the survey completed all questions, resulting in a 100% completion rate. | Research Design |
| Number of screens (pages) | The questionnaire consisted of a total of 21 pages. | Research Design |
| Completeness check | All questions were set as mandatory. Participants could not proceed to the next page without answering all questions, and the system was designed to highlight any unanswered fields in real time to prompt users to fill them in. | Research Design |
| Review step | Respondents were able to use the “Back” button during the survey to review and change their answers. However, they were unable to make changes after the survey was submitted. | Research Design |
| Unique site visitor | Regarding unique site visitors, the data retention period for the survey system ORCA had expired, which made calculation impossible. Therefore, this information has not been included. | None |
| View rate (Ratio of unique survey visitors/unique site visitors) | The data retention period for the ORCA survey system had expired, and the number of unique visitors to the first page of the survey is unknown. Therefore, the view rate could not be ascertained. | None |
| Participation rate (Ratio of unique visitors who agreed to participate/unique first survey page visitors) | The participation rate was 57.3%. | Participant Characteristics |
| Completion rate (Ratio of users who finished the survey/users who agreed to participate) | Of the 1,095 individuals targeted, 350 completed the survey, resulting in a final response rate of 31.9% (350/1,095). As all questions in this survey were mandatory, there were no partial responses, and the attrition rate was 0%. | Participant Characteristics |
| Cookies used | No use of cookies. | None |
| IP check | Use of IP address is available. | Targeting and Survey Distribution |
| Log file analysis | No use of log file analysis. | None |
| Registration | Because this was a private survey, users were required to log in to the site, which made duplicate responses from the same user impossible within the system. Once a user entered the survey, they were unable to enter it again. | Targeting and Survey Distribution |
| Handling of incomplete questionnaires | Only completed questionnaires were included in the analysis. | None |
| Questionnaires submitted with an atypical timestamp | We did not measure response times. | None |
| Statistical correction | We did not perform any statistical adjustments, such as weighting or propensity score adjustments, after the survey. Regarding representativeness, a 1:1 allocation for sex was implemented during the survey design phase, which resulted in 175 men and 175 women, totaling 350 participants in the study. | Targeting and Survey Distribution |

This checklist has been modified from Eysenbach G. Improving the quality of Web surveys: the Checklist for Reporting Results of Internet E-Surveys (CHERRIES). J Med Internet Res. 2004 Sep 29; September):e34 [erratum in J Med Internet Res. 2012; 14(1): e8.]. Article available at https://www.jmir.org/2004/3/e34/; erratum available https://www.jmir.org/2012/1/e8/. Copyright Gunther Eysenbach. Originally published in the Journal of Medical Internet Research, 29.9.2004 and 04.01.2012.

This is an open-access article distributed under the terms of the Creative Commons Attribution License (https://creativecommons.org/licenses/by/2.0/), which permits unrestricted use, distribution, and reproduction in any medium, provided the original work, first published in the Journal of Medical Internet Research, is properly cited.
